# Repeated Administration of Clinically Relevant Doses of the Prescription Opioids Tramadol and Tapentadol Causes Lung, Cardiac, and Brain Toxicity in Wistar Rats

**DOI:** 10.3390/ph14020097

**Published:** 2021-01-27

**Authors:** Joana Barbosa, Juliana Faria, Fernanda Garcez, Sandra Leal, Luís Pedro Afonso, Ana Vanessa Nascimento, Roxana Moreira, Frederico C. Pereira, Odília Queirós, Félix Carvalho, Ricardo Jorge Dinis-Oliveira

**Affiliations:** 1 IINFACTS—Institute of Research and Advanced Training in Health Sciences and Technologies, Department of Sciences, University Institute of Health Sciences (IUCS), CESPU, CRL, 4585-116 Gandra, Portugal; juliana.faria@iucs.cespu.pt (J.F.); fernanda.garcez@cespu.pt (F.G.); sandra.leal@iucs.cespu.pt (S.L.); anavanessa65@gmail.com (A.V.N.); roxanamoreira@moreno.pt (R.M.); odilia.queiros@iucs.cespu.pt (O.Q.); 2 UCIBIO, REQUI*M*TE—Laboratory of Toxicology, Department of Biological Sciences, Faculty of Pharmacy, University of Porto, 4050-313 Porto, Portugal; felixdc@ff.up.pt; 3 Department of Public Health and Forensic Sciences, and Medical Education, Faculty of Medicine, University of Porto, 4200-319 Porto, Portugal; 4 Department of Biomedicine, Unit of Anatomy, Faculty of Medicine, University of Porto, 4200-319 Porto, Portugal; 5 CINTESIS—Center for Health Technology and Services Research, Faculty of Medicine, University of Porto, 4200-450 Porto, Portugal; 6 Department of Pathology, Portuguese Institute of Oncology of Porto, 4200-072 Porto, Portugal; lpafonso@gmail.com; 7 Institute of Pharmacology and Experimental Therapeutics/iCBR, Faculty of Medicine, University of Coimbra, 3000-354 Coimbra, Portugal; fredcp@ci.uc.pt

**Keywords:** tramadol, tapentadol, prescription opioids, pneumotoxicity, cardiotoxicity, neurotoxicity, in vivo studies

## Abstract

Tramadol and tapentadol, two structurally related synthetic opioid analgesics, are widely prescribed due to the enhanced therapeutic profiles resulting from the synergistic combination between μ-opioid receptor (MOR) activation and monoamine reuptake inhibition. However, the number of adverse reactions has been growing along with their increasing use and misuse. The potential toxicological mechanisms for these drugs are not completely understood, especially for tapentadol, owing to its shorter market history. Therefore, in the present study, we aimed to comparatively assess the putative lung, cardiac, and brain cortex toxicological damage elicited by the repeated exposure to therapeutic doses of both prescription opioids. To this purpose, male Wistar rats were intraperitoneally injected with single daily doses of 10, 25, and 50 mg/kg tramadol or tapentadol, corresponding to a standard analgesic dose, an intermediate dose, and the maximum recommended daily dose, respectively, for 14 consecutive days. Such treatment was found to lead mainly to lipid peroxidation and inflammation in lung and brain cortex tissues, as shown through augmented thiobarbituric acid reactive substances (TBARS), as well as to increased serum inflammation biomarkers, such as C reactive protein (CRP) and tumor necrosis factor-α (TNF-α). Cardiomyocyte integrity was also shown to be affected, since both opioids incremented serum lactate dehydrogenase (LDH) and α-hydroxybutyrate dehydrogenase (α-HBDH) activities, while tapentadol was associated with increased serum creatine kinase muscle brain (CK-MB) isoform activity. In turn, the analysis of metabolic parameters in brain cortex tissue revealed increased lactate concentration upon exposure to both drugs, as well as augmented LDH and creatine kinase (CK) activities following tapentadol treatment. In addition, pneumo- and cardiotoxicity biomarkers were quantified at the gene level, while neurotoxicity biomarkers were quantified both at the gene and protein levels; changes in their expression correlate with the oxidative stress, inflammatory, metabolic, and histopathological changes that were detected. Hematoxylin and eosin (H & E) staining revealed several histopathological alterations, including alveolar collapse and destruction in lung sections, inflammatory infiltrates, altered cardiomyocytes and loss of striation in heart sections, degenerated neurons, and accumulation of glial and microglial cells in brain cortex sections. In turn, Masson’s trichrome staining confirmed fibrous tissue deposition in cardiac tissue. Taken as a whole, these results show that the repeated administration of both prescription opioids extends the dose range for which toxicological injury is observed to lower therapeutic doses. They also reinforce previous assumptions that tramadol and tapentadol are not devoid of toxicological risk even at clinical doses.

## 1. Introduction

Opioids currently represent a mainstay option for the treatment of moderate to severe forms of pain. In this context, tramadol and tapentadol, synthetic and structurally related opioids, are widely prescribed in acute and chronic settings, finding application in the treatment of several clinical conditions, such as postoperative, musculoskeletal, neuropathic, cancer and mixed pain states [1,2,3,4,5,6,7,8,9,10,11,12]. However, the misuse and abuse of prescription opioids, such as tramadol and tapentadol, is increasing due to their easy access, leading to addiction and toxicity cases. Thus, understanding the toxicology of prescription opioids is a challenge for modern societies.

Tramadol (1*RS*, 2*RS*)-2-[(dimethylamino)methyl]-1-(3-methoxyphenyl)-cyclo-hexanol) is a racemic opioid [13,14], whose analgesic efficiency is dependent on its metabolization to *O*-desmethyltramadol (M1) via cytochrome P450 (CYP450) [13,14,15,16,17]. In turn, tapentadol, 3-[(1*R*,2*R*)-3-(dimethylamino)-1-ethyl-2-methylpropyl]phenol, is a single active molecule. Both opioids combine μ-opioid receptor (MOR) activation and serotonin (5-HT) and noradrenaline (NA) reuptake inhibition [13,14], although tapentadol shows minimal 5-HT reuptake inhibition properties [14,17,18,19,20,21,22,23]. Interestingly, tapentadol noradrenergic component is associated with anti-apoptotic and pro-neurogenic effects, counteracting MOR-mediated deleterious effects. This protective effect, along with the increasing contribution of the noradrenergic component in persistent neuropathic states, supports its use in neuropathic pain treatment [1,4,5,6,24,25,26,27].

Although tramadol and tapentadol are safe and effective in pain relief, they have already been associated with many cases of addiction and toxicity, some of which fatal [1,9,28,29,30,31,32,33,34,35,36,37,38,39,40,41,42,43,44,45,46,47,48,49,50,51,52,53]. Such observations emphasize the importance of understanding the mechanisms underlying their toxicity. Our group has already studied the effects of an acute exposure to clinically relevant doses of tramadol and tapentadol [54,55,56]. We previously reported biochemical alterations in serum and urine samples from in vivo models, as well as in a neuronal cell model, having found oxidative status and histological alterations in brain cortex, lung, heart, liver and kidney tissues [54,55,56]. Our results showed that, in acute contexts, tapentadol causes more pronounced toxic damage [54,55,56]. In a more recent study by our group, we showed that repeated administration of clinically relevant doses of tramadol and tapentadol smooths the differences between the toxicological profiles of both opioids, and that hepatorenal damage occurs at lower doses, when compared with acute exposure [57]. Several other studies with animal models were performed with high tramadol doses, in particular the median lethal dose (LD_50_). In the rat model, tramadol LD_50_ was already associated with brain congestion, edema, gliosis, microglial and oligodendrocyte proliferation and inflammatory cell infiltrates [58], while its repeated administration at doses ranging from 30 to 168 mg/kg induced several brain and lung histological alterations [58,59,60,61,62]. Besides histological changes, chronic tramadol administration in rodents was also associated with increased reactive oxygen species (ROS) and mitochondrial alterations in tissues such as brain and lung [58,59,63]. In fact, treatment with antioxidants is suggested as a strategy to decrease tramadol-induced tissue damage [64]; prolonged dose interval or dose reductions are also suggested during chronic treatment [65].

Concerning tapentadol toxicity, Channell and Schug reported many adverse events, including neurological, respiratory, and cardiac function impairment [29]. However, few studies were performed to understand the mechanisms associated with tapentadol toxicity, as underlined in their systematic review [29], since it is a more recent drug. In addition, there are few comparative studies on the long-term effects of clinical doses of tramadol or tapentadol [57], particularly in target tissues such as brain, heart, and lung.

Hence, the present work aimed to evaluate the in vivo toxicological effects of the repeated administration of clinically relevant doses of tramadol or tapentadol, through the comparative analysis of brain, cardiac and lung toxicity. Our study combines molecular, biochemical, and histological approaches and, thus, contributes to a more complete and comprehensive understanding of tramadol and tapentadol toxicological profile.

## 2. Results

### 2.1. Repeated Exposure to Tramadol and Tapentadol Causes Oxidative Stress in Lung and Brain Cortex

**Figure 1:**
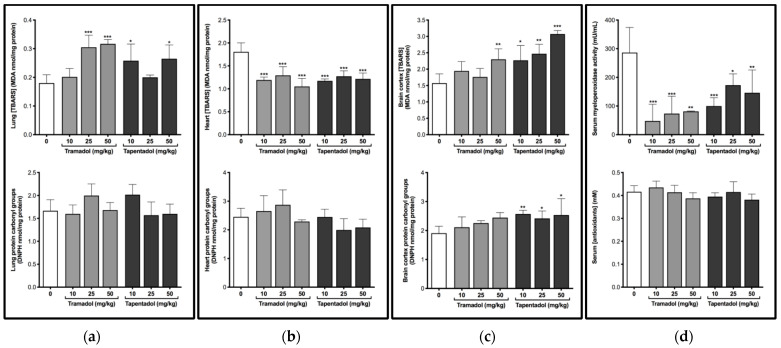
Oxidative stress analysis, assayed as thiobarbituric acid reactive substances (TBARS) and protein carbonyl groups, in Wistar rat lung (**a**), heart (**b**) and brain cortex (**c**) tissue homogenates, as well as serum myeloperoxidase (MPO) activity and total antioxidant capacity (Trolox equivalents) (**d**). Both tissue homogenates and serum samples were processed upon repeated daily intraperitoneal (i.p.) administration of 10, 25, or 50 mg/kg tramadol or tapentadol, for 14 consecutive days. TBARS and protein carbonyl group results were normalized against total protein content. Results are expressed by means ± SD. *** *p* < 0.001, ** *p* < 0.01, * *p* < 0.05. DNPH: 2,4-dinitrophenylhydrazine; MDA: malondialdehyde.

Wistar rats were used as a model to study the effect of repeated administration of tramadol and tapentadol in lung, heart, and brain cortex. In order to evaluate the effects on oxidative status and putative oxidative damage, thiobarbituric acid reactive substances (TBARS), protein carbonyl groups, myeloperoxidase (MPO) activity and total antioxidant capacity were quantified in tissue and serum samples (Figure 1).

A significant increase in lung TBARS levels was observed after exposure to 25 and 50 mg/kg tramadol (rising around 1.7-fold), and 10 and 50 mg/kg tapentadol (rising around 1.5-fold) (Figure 1a). In turn, in heart tissue, TBARS levels decreased to about 67% of the control, on average, at all doses of both opioids (Figure 1b). Analysis of brain cortex homogenates showed that the highest tramadol dose, 50 mg/kg, causes a significant 1.5-fold increase in TBARS levels, while this happened for all tapentadol doses (around 1.7-fold, on average) (Figure 1c). No significant differences were observed for protein carbonyl groups in any of the organs studied, except for brain cortex at all tapentadol doses, for which they increased about 1.3-fold, on average (Figure 1c). These results suggest that, among the tissues under analysis, brain cortex is more susceptible to oxidative damage, particularly after tapentadol exposure. Regarding serum MPO activity, a significant decrease was observed after exposure to both opioids, and at all doses tested, with the values reaching about 36% of the control, on average (Figure 1d). Nonetheless, the exposure to tramadol or tapentadol did not lead to alterations in serum total antioxidant capacity (Figure 1d).

### 2.2. Repeated Exposure to Tramadol and Tapentadol Causes Alterations in Immunological and Inflammatory Biomarkers

**Figure 2:**
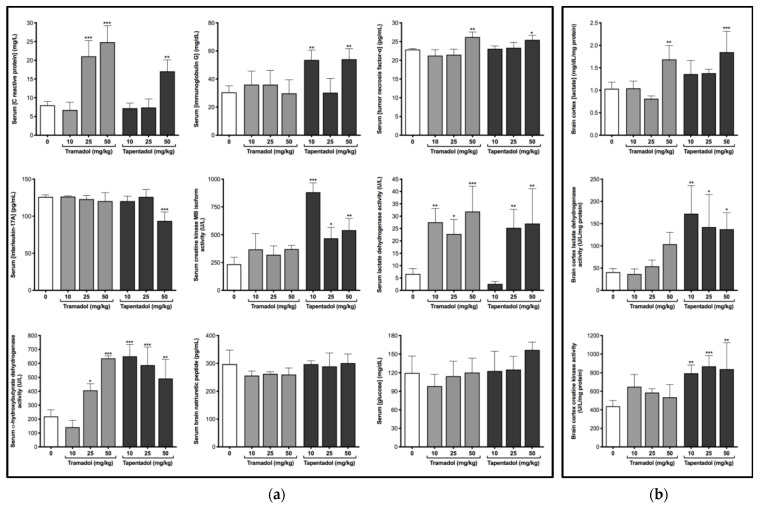
Concentrations of serum immunological, inflammatory, cardiac and metabolic biomarkers (**a**), as well as tissue biochemical parameters concerning brain cortex metabolism (**b**), upon Wistar rat repeated daily intraperitoneal (i.p.) administration of 10, 25, or 50 mg/kg tramadol or tapentadol, for 14 consecutive days. Results are expressed as means ± SD. *** *p* < 0.001, ** *p* < 0.01, * *p* < 0.05.

Aiming to evaluate the effects of the repeated administration of therapeutic doses of tramadol and tapentadol on the immunological and inflammatory status, some serum biomarkers were tested, as shown in Figure 2a.

Exposure to 25 and 50 mg/kg tramadol led to an increase in C reactive protein (CRP) levels (2.9-fold, on average); the highest tramadol dose also caused a significant increase in tumor necrosis factor-α (TNF-α) levels (1.2-fold). 50 mg/kg tapentadol led to an increase in CRP (2.1-fold) and TNF-α (1.1-fold). In turn, immunoglobulin G (IgG) levels increased about 1.8-fold, on average, at tapentadol lowest and highest doses. Although no effects were detected on interleukin-17A (IL-17A) levels after tramadol exposure, they significantly decreased at 50 mg/kg tapentadol, reaching 74% of the control values.

### 2.3. Repeated Exposure to Tramadol and Tapentadol Compromises Cardiac Cell Integrity and Brain Cortex Metabolism

Several serum biomarkers were analyzed in order to evaluate cardiac cell integrity and function, as shown in Figure 2a. While creatine kinase muscle brain (CK-MB) isoform activity did not change significantly upon tramadol treatment, lactate dehydrogenase (LDH) activity significantly increased at all its doses, rising around 4.1-fold, on average, above the control. However, α-hydroxybutyrate dehydrogenase (α-HBDH) activity increased only when the intermediate and highest doses of tramadol (25 and 50 mg/kg) were administered, to a maximum of 2.9-fold. In turn, 25 and 50 mg/kg tapentadol doses led to an approximate increase of 3.9-fold in LDH activity. At all doses tested, tapentadol caused an increase in CK-MB (to a maximum of 3.8-fold) and α-HBDH (2.6-fold, on average) activities. Though serum brain natriuretic peptide (BNP) levels did not change significantly after exposure to any of the opioid doses tested, when taken together, these data suggest that tramadol and tapentadol cause cardiac damage.

The analysis of biochemical parameters related to brain cortex metabolism (Figure 2b) showed 50 mg/kg tramadol and tapentadol to cause a significant increase (1.6- and 1.8-fold, respectively) in tissue lactate levels. Contrarily to tapentadol, which caused an increase in brain LDH and creatine kinase (CK) activities (3.7-fold and 1.9-fold, on average, respectively), irrespectively of the dose, tramadol led to no statistically significant differences in these enzymes. Serum glucose concentrations also did not change in a significant manner (Figure 2a). These results collectively suggest that tapentadol causes higher brain metabolic alterations.

### 2.4. Repeated Exposure to Tramadol and Tapentadol Leads to Changes in the Expression of Lung, Heart and Brain Toxicity Biomarkers

**Figure 3:**
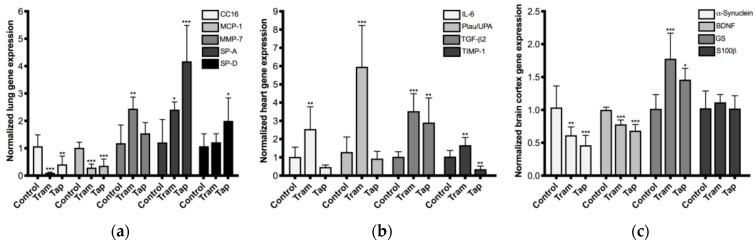
Normalized gene expression levels of lung (**a**), heart (**b**) and brain cortex (**c**) toxicity biomarkers, upon Wistar rat repeated daily intraperitoneal (i.p.) administration of 50 mg/kg tramadol (Tram) or tapentadol (Tap), for 14 consecutive days. Expression levels were normalized against the respective 18S ribosomal RNA (18S rRNA) gene expression, and then against the respective controls (administered with normal saline), set as 1. Results are expressed as means ± SD. *** *p* < 0.001, ** *p* < 0.01, * *p* < 0.05. BDNF: brain-derived neurotrophic factor; CC16: Clara cell protein-16; GS: glutamine synthetase; IL-6: interleukin-6; MCP-1: monocyte chemoattractant protein-1; MMP-7: matrix metalloproteinase-7; Plau/UPA: plasminogen activator, urokinase; S100β: S100 calcium binding protein B; SP-A: pulmonary surfactant protein A; SP-D: pulmonary surfactant protein D; TGF-β2: transforming growth factor-β2; TIMP-1: tissue inhibitor of metalloproteinase-1.

**Figure 4:**
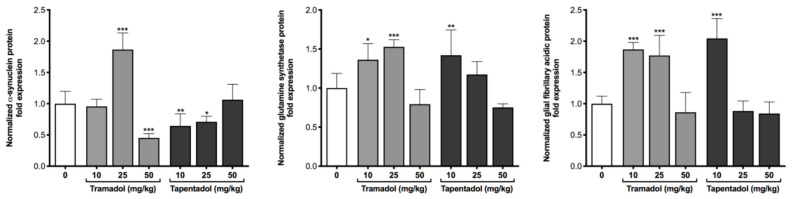
Normalized protein expression levels of neuronal marker α-synuclein and astrocytic markers glutamine synthetase (GS) and glial fibrillary acidic protein (GFAP), in protein extracts from brain cortex tissue, upon Wistar rat repeated daily intraperitoneal (i.p.) administration of 10, 25 or 50 mg/kg tramadol or tapentadol, for 14 consecutive days. Expression levels were normalized against total protein content, using α-tubulin as loading control, and then against the respective controls (administered with normal saline), set as 1. Results are expressed as means ± SD. *** *p* < 0.001, ** *p* < 0.01, * *p* < 0.05.

Potential toxic effects arising from the repeated administration of clinically relevant doses of tramadol and tapentadol were investigated at the molecular level, through the quantification of toxicity biomarker genes and proteins in lung, heart and brain cortex tissue samples. To this purpose, total RNA from tissues collected from animals exposed to 50 mg/kg tramadol or tapentadol were used in gene expression assays (Figure 3). In turn, brain cortex extracts from animals treated with all opioid doses were used in protein expression assays of neuronal and astrocytic markers (Figure 4).

Five pulmonary toxicity biomarkers were analyzed, as shown in Figure 3a. Tramadol and tapentadol caused a significant decrease in the expression of Clara cell protein-16 (CC16, reaching 10% and 41% of the control, respectively) and monocyte chemoattractant protein-1 (MCP-1, achieving 29% and 36% of the control, respectively). On the other hand, tramadol led to a significant increase in the expression of matrix metalloproteinase-7 (MMP-7, 2.4-fold), tapentadol caused an increase in the expression of pulmonary surfactant protein D (SP-D, 2.0-fold), and both increased the expression of pulmonary surfactant protein A (SP-A, whose gene expression increased 2.4-fold and 4.2-fold upon exposure to 50 mg/kg tramadol and tapentadol, respectively). Concerning cardiac biomarkers (Figure 3b), tramadol caused an increase in the expression of interleukin-6 (IL-6, 2.5-fold) and plasminogen activator, urokinase (Plau/UPA, 6.0-fold); tramadol and tapentadol increased transforming growth factor-β2 (TGF-β2) expression (3.5-fold and 2.9-fold, respectively). Regarding tissue inhibitor of metalloproteinase-1 (TIMP-1) expression, tramadol caused an increase (1.7-fold), unlike tapentadol, which induced a considerable reduction (achieving 35% of the control). The results from brain biomarker gene analysis (Figure 3c) showed that the exposure to tramadol and tapentadol causes a decrease in the expression of α-synuclein (to about 62% and 46% of the control values, respectively) and brain-derived neurotrophic factor (BDNF, reaching 78% and 68% of the control, respectively), as well as an increase in glutamine synthetase (GS) expression (1.8-fold and 1.5-fold, respectively). Nonetheless, no significant differences in S100 calcium binding protein B (S100β) levels were found after tramadol or tapentadol treatment.

As shown in Figure 4, the protein expression levels of a neuronal marker (α-synuclein) and two astrocytic markers (GS and glial fibrillary acidic protein (GFAP)) are altered upon treatment with both opioids. The 25 mg/kg tramadol dose increased the levels of neuronal marker α-synuclein by 1.9-fold; in contrast, the 50 mg/kg dose induced its decrease (achieving 45% of the control levels). The exposure to 10 and 25 mg/kg tramadol caused a significant increase in GS and GFAP protein levels (average 1.4- and 1.8-fold, respectively). The treatment with 10 mg/kg tapentadol caused a significant increase in GS and GFAP protein levels (1.4- and 2.0-fold, respectively), while 10 and 25 mg/kg tapentadol caused a decrease in the neuronal marker α-synuclein (to about 64% and 71% of the control values, respectively).

Taken together, such results demonstrate that the repeated administration of tramadol and tapentadol clinically relevant doses impacts lung, heart, and brain cortex physiology and metabolism at the gene and protein levels.

### 2.5. Repeated Exposure to Tramadol and Tapentadol Leads to Lung Alveolar Collapse, Cardiac Inflammation and Fibrosis and Neuronal Degeneration

**Figure 5:**
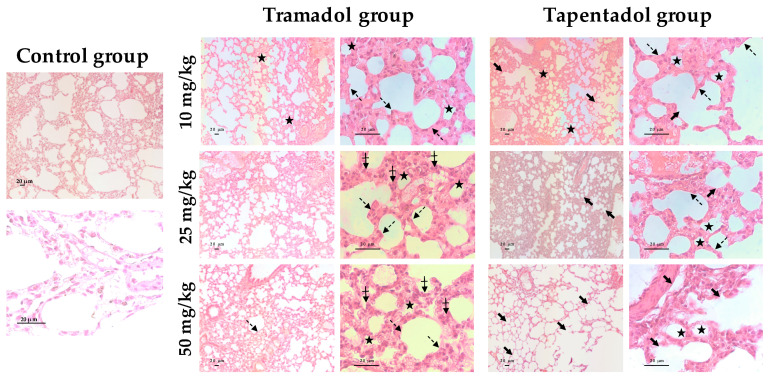
Photomicrographs of lung sections of Wistar rats intraperitoneally injected with different tramadol and tapentadol doses or saline (control group), for 14 consecutive days, upon hematoxylin and eosin (H & E) staining. Alveolar collapse (stars), alveolar wall thickening and hyperpigmentation (dashed arrows) and disorganized cells (vertical, crossed arrows), as well as alveolar destruction and loss of parenchyma (thick arrows), are observed. Photographs were taken with 100× and 600× magnifications. Scale bar, 20 μm.

**Figure 6:**
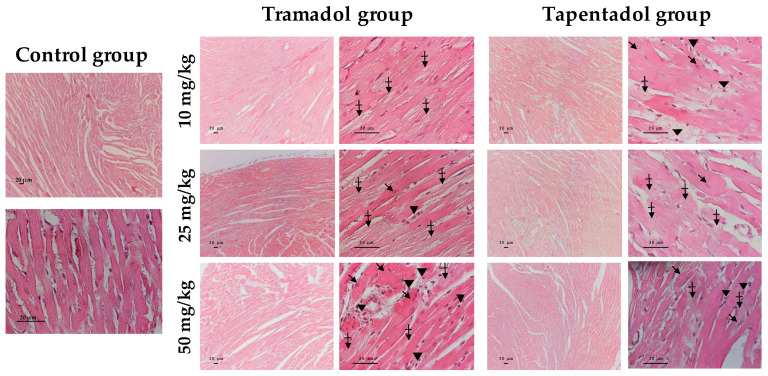
Photomicrographs of heart sections of Wistar rats intraperitoneally injected with different tramadol and tapentadol doses or saline (control group), for 14 consecutive days, upon hematoxylin and eosin (H & E) staining. A dotted staining (vertical, crossed arrows), possibly denoting fibrous tissue deposition, is observed among cardiomyocytes. Mononuclear inflammatory cells (inverted triangles) and altered cardiomyocytes (arrows), as well as loss of striation, are also observed. Photographs were taken with 100× and 600× magnifications. Scale bar, 20 μm.

**Figure 7:**
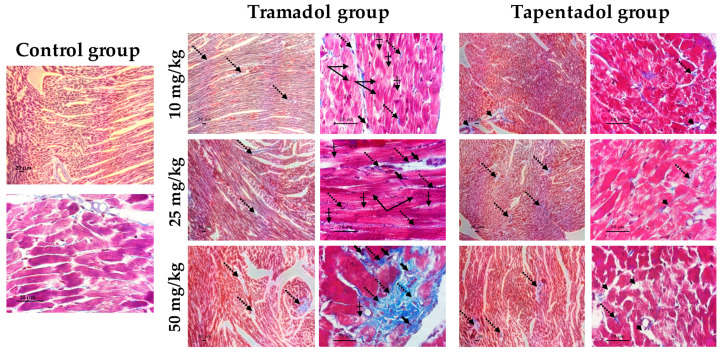
Photomicrographs of heart sections of Wistar rats intraperitoneally injected with different tramadol and tapentadol doses or saline (control group), for 14 consecutive days, upon Masson’s trichrome staining. Fibrous tissue (dotted arrows) and purple cell infiltrates (thick arrows), possibly corresponding to fibroblasts, as well as a dotted staining (vertical, crossed arrows), are observed among cardiomyocytes. Cardiomyocyte fiber filaments are disorganized and show heterogeneous pigmentation (double arrows). Increased perivascular spaces (arrow heads) are also observed. Photographs were taken with 100× and 600× magnifications. Scale bar, 20 μm.

**Figure 8:**
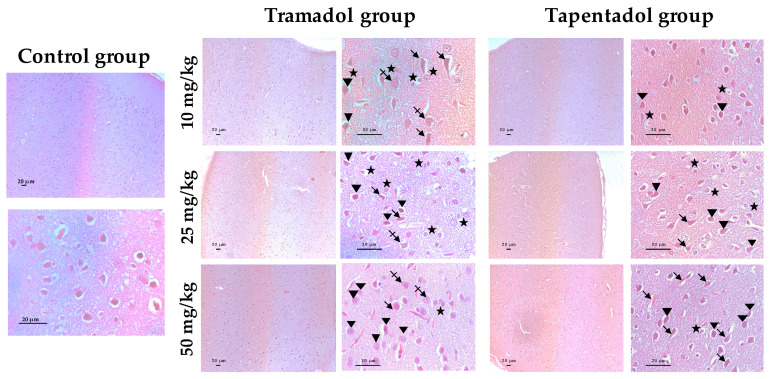
Photomicrographs of brain cortex sections of Wistar rats intraperitoneally injected with different tramadol and tapentadol doses or saline (control group), for 14 consecutive days, upon hematoxylin and eosin (H & E) staining. Glial and microglial cells are observed (stars), as well as swollen neurons (crossed arrows), irregularly-shaped neurons (inverted triangles) and degenerated neurons (long arrows). Photographs were taken with 100× and 600× magnifications. Scale bar, 20 μm.

Putative histopathological alterations induced by the treatment with tramadol or tapentadol therapeutic doses were also investigated in lung (Figure 5), heart (Figure 6 and Figure 7), and brain cortex (Figure 8) tissues.

The histological study of lung tissue samples, upon hematoxylin and eosin (H & E) staining (Figure 5), showed alveolar collapse and wall thickening, as well as hyperpigmentation, to be consequences of the exposure to all tramadol and tapentadol doses. At higher tramadol doses (25 and 50 mg/kg), disorganized cells were observed. Additionally, after tapentadol treatment, alveolar destruction and loss of parenchyma were observed, leading to a “holey” pattern, even in the vicinity of great vessels.

Figure 6 evidences heart tissue damage caused by tramadol and tapentadol, as seen through H & E staining. Interestingly, treatment with tramadol comparatively led to more pronounced injury along with dose increase. A dotted staining between cardiomyocytes, possibly reflecting cardiomyocyte substitution by fibrous tissue, is observed as a consequence of tramadol treatment. Furthermore, at higher doses (25 and 50 mg/kg), mononuclear inflammatory cells and altered cardiomyocytes were detected, as well as loss of striation. At the highest dose (50 mg/kg), a lower pigmentation was observed in vessel vicinity, suggesting a context of perivascular fibrosis. In turn, tapentadol caused similar alterations at all doses, including the dotted staining between cardiomyocytes, eventually suggesting fibrous tissue deposition; inflammatory cell infiltrates, altered cardiomyocytes and loss of striation were also found.

In order to clarify the potential signs of fibrosis suggested by H & E staining, Masson’s trichrome staining was performed with heart tissue samples (Figure 7).

Indeed, the presence of fibrous tissue between cardiomyocytes was confirmed after tramadol and tapentadol treatment, being evident at tramadol doses as low as 10 mg/kg; such observations increased along with dose increment, with the 50 mg/kg dose leading to marked perivascular fibrosis. Besides these findings, cardiomyocyte fiber filaments are disorganized and show heterogeneous pigmentation. In animals treated with tapentadol, fibrous tissue was observed between cardiomyocytes, possibly delimiting newly formed capillaries and, thereby, suggesting revascularization. Despite being found at all tapentadol doses, a more evident increase in perivascular space was observed at 50 mg/kg.

Brain cortex histological analysis through H & E staining is shown in Figure 8. Both tramadol and tapentadol exposure cause glial activation with microglial proliferation and are associated with degenerated and irregularly-shaped neurons. Such histopathological changes accumulated along with tapentadol dose. Moreover, tramadol treatment led to swollen neurons.

Thus, clinically relevant doses of both tramadol and tapentadol lead to histopathological damage in all tissues under analysis.

## 3. Discussion

Prescription opioids are not exempt from toxicological risks, especially when used for prolonged periods. In the present study, we aimed to analyze putative lung, heart, and brain cortex detrimental effects deriving from the repeated administration of clinically relevant doses of tramadol and tapentadol to Wistar rats. By addressing, in parallel, tramadol and tapentadol subacute effects on target organs, we complement the subacute study regarding the effects on metabolizing organs, as well as our previous acute exposure studies. In fact, since these opioids are often consumed on a subacute to chronic basis, our repeated exposure-based experimental design also provides a realistic approximation to their actual consumption scenario.

Comparison of tramadol and tapentadol safety profiles is justified by their structural and mechanistic similarities. However, when comparing their toxicological effects, differences in their pharmacokinetic and pharmacodynamic properties, including metabolic pathways, metabolite profiles, receptor, and transporter affinities [1,9,55,56,57], should be kept in mind. Additionally, intraperitoneally-injected drugs bypass the intestine, but are absorbed into the mesenteric vessels draining into the portal vein, thereby giving room for hepatic metabolism to occur before reaching systemic circulation [66]. Hence, there are pharmacokinetic similarities between intraperitoneal (i.p.) and oral administration, which is a common route of administration, and the only one in which tapentadol is currently available [1,9]. Thus, although the doses used in our study are equal in absolute terms, they are not pharmacologically equivalent. In fact, the two opioids present different oral bioavailabilities (68–84% for tramadol and 32% for tapentadol [1,9,13]). In line with this, tapentadol doses should be increased to achieve pharmacological equivalence to tramadol, which would furthermore exacerbate differences between the results. All these remarks should be taken into account while comparatively addressing both toxicological profiles.

### 3.1. Repeated Administration of Tramadol and Tapentadol Leads Mainly to Lipid Peroxidation in Lung and Brain Cortex Tissues, but Has Seemingly a Protective Effect in Cardiac Tissue

Although their dose determines their overall effect on the oxidation status, opioids are known to induce oxidative stress, with multiple studies reporting increased serum and tissue oxidative stress biomarkers and decreased antioxidant defense mechanisms. Decreased brain glutathione, glutathione peroxidase, and superoxide dismutase (SOD) activities, as well as increased brain malondialdehyde (MDA), nitric oxide (NO), inducible nitric oxide synthase (iNOS), and 8-hidroxydeoxyguanosine levels, have been described in mice and rat models repeatedly administered with 20 to 168 mg/kg tramadol, through different routes [60,62,67,68,69,70,71]. Furthermore, under the conditions assayed in the present study, we have previously shown increased TBARS—a surrogate of lipid peroxidation (LPO)—and protein carbonyl groups—indicative of protein oxidation—in liver and kidney homogenates from Wistar rats exposed to both tramadol and tapentadol [57].

In single-exposure assays to 10, 25 and 50 mg/kg tramadol and tapentadol, we found almost no significant alterations in TBARS levels in lung, heart, and brain cortex homogenates [55]. In turn, the protein carbonyl group contents increased in lung and heart tissues at the intermediate and highest doses, whilst they significantly decreased in brain cortex upon tramadol treatment [55]. Repeated administration changed such scenario, since, following exposure to both opioids, TBARS concentrations increased in lung and brain cortex, while they decreased in heart homogenates (Figure 1). Protein carbonyl groups did not change significantly, except for brain cortex from animals exposed to tapentadol, where they increased (Figure 1c).

Therefore, it might be hypothesized that prolonged administration changes the oxidation status in an opioid- and organ-specific manner. Indeed, LPO was now induced in lung and brain cortex, but there seems to be a protective effect in heart tissue. Consistently with this, 20 mg/kg tramadol prevented a rise in cardiac tissue MDA levels in a rat ischemia-reperfusion model [72]. The authors of the study suggest that tramadol reduces oxidative stress by scavenging peroxyl radicals and increasing antioxidant capacity [72]. In this regard, serum MPO results should also be taken into account. MPO is a member of the superfamily of heme peroxidases that is mainly expressed in polymorphonuclear neutrophils and monocytes, which contribute to the generation of reactive species that elicit inflammation and LPO [73]. Several lines of evidence support an association between MPO (and its product hypochlorous acid (HOCl)) and cardiovascular disease, given that, among other effects, it generates dysfunctional lipoproteins and atherosclerotic plaque instability [73]. Hence, reduced MPO activity might be correlated with cardiac tissue protection from LPO. In fact, MPO activity was found to be reduced in lung tissue, relative to the controls, after intravenous administration of 20 mg/kg tramadol in a rat model of ischemia-reperfusion [74]. Interestingly, morphine has been reported as an MPO inhibitor [75], for which a similar effect might be anticipated for other structurally related opioids. Still, tissue quantification of MPO activity would add information on this aspect, since an increase in its levels was reported in rat brain tissue following a 9-week daily treatment with 22.5 to 90 mg/kg tramadol [68].

As far as protein carbonyl groups are concerned, the protective effect observed in brain samples in an acute context is lost upon repeated tapentadol administration, while the deleterious effects in lung and heart appear to fade for both opioids.

While tramadol effects on brain cortex oxidative stress are minor, they are more substantial upon tapentadol treatment. In fact, due to its much greater potency at the MOR, comparable or higher NA transporter inhibition and significantly lower 5-HT transporter inhibition, tapentadol has a greater central nervous system (CNS) functional activity than tramadol, being 2 to 5 times more potent across different animal models of pain [14]. In addition, in vitro and in vivo studies suggest that tramadol is actively transported across the blood-brain barrier, at least in part, by proton-coupled organic cation antiporter [76]. Disproportionally less of the stronger opioid metabolite M1 crosses the blood-brain barrier than its weaker opioid parent, with the disparity increasing as tramadol dose is increased [14]. In turn, tapentadol readily crosses the blood-brain barrier, following its concentration gradient, with no known active transport mechanism [77]. Altogether, these pharmacokinetic and pharmacodynamic differences may contribute to explain the greater impact of tapentadol on the CNS and, more specifically, on brain cortex oxidative stress. The reasons underlying tapentadol higher potency and efficacy may simultaneously underlie its deleterious effects in target organs.

Taken together, the results indicate that the extension of the exposure period leads to a shift towards the intensification of lipid oxidative stress mechanisms, from which the cardiac tissue seems to be spared. The results obtained with brain samples might be correlated with the high rates of brain oxygen consumption, which make it particularly prone to oxidative damage. Nonetheless, such local, organ-specific alterations do not impact the systemic antioxidant status, since no significant alterations were found in the serum concentration of antioxidants. It should be noted, however, that, although total antioxidant capacity assays predominantly measure low molecular weight, chain breaking antioxidants such as urate, ascorbate, bilirubin, and α-tocopherol, they do not measure important antioxidant components such as SOD, glutathione peroxidase, and catalases [78,79]. While the serum levels of the former, in opioid-treated rats, might be comparable to those of the controls—as deduced from the absence of statistically significant differences among groups, the activities of the latter might be decreased, both in serum and in tissues. This possibly explains increased tissue lipid and protein oxidative stress and is supported by several studies reporting decreased antioxidant enzyme activity upon tramadol exposure [60,67,68,69,71].

### 3.2. Repeated Administration of Tramadol and Tapentadol Leads to Inflammation, with Possible Compensatory Recruitment of Anti-Inflammatory Pathways

Opioids are suggested to suppress immune competency in pain-free subjects, even at subanalgesic doses [80]. In fact, tramadol has been reported to have anti-inflammatory properties [81] and to lead to less immunomodulatory effects when compared with pure MOR agonists, which are known for suppressing natural killer (NK) cell activity and T lymphocyte proliferation [80]. Nonetheless, diverse histopathological studies, some of which by our own group [55,56,57], describe its ability, as well as that of tapentadol, to cause tissue inflammation in acute and subacute contexts. In order to understand if a subacute exposure to tramadol and tapentadol clinically relevant doses causes immunological and inflammatory alterations, some serum biomarkers were analyzed (Figure 2a).

CRP is an acute-phase protein, synthesized by the liver, whose plasma levels increase in response to inflammation. Our results show that serum levels of CRP increase after tramadol and tapentadol administration, which is compatible with an inflammatory condition. Consistently, patients who received 100 mg tramadol every 8 h experienced a 123%-increase over their CRP baseline, 72 h after removal of an impacted lower third molar [81]. Moreover, following the administration of the highest dose in the present study (50 mg/kg), we also detected an increase in TNF-α, a cytokine involved in both physiological and pathological processes. Due to its participation in essential cellular pathways associated with inflammation, apoptosis, and necrosis, it is used as a systemic marker for tissue injury and systemic inflammation [68]. A significant increase in serum concentrations of pro-inflammatory cytokines TNF-α and interleukin-1B (IL-1B) had already been associated with chronic administration of therapeutic (22.5 mg/kg/day) and high tramadol doses (30, 60, and 90 mg/kg/day) [68]. Increased serum IgG levels were also found upon exposure to tapentadol. Both pro- and anti-inflammatory roles have been associated with IgG, the most abundant antibody in human serum and an indicator of the immune status [82]. In particular, raised IgG serum concentrations are found in interstitial lung disease, characterized by chronic inflammation and irritation of the alveolar walls (alveolitis) and adjacent supporting tissue (interstitium), which may progress to fibrosis [83], and are thus compatible with the histopathological alterations observed in lung slides (Figure 5). Interestingly, elevated serum IgG levels were found at 10 and 50 mg/kg tapentadol, but not at 25 mg/kg. We hypothesize that both the lowest and highest tapentadol doses lead to an inflammatory state, characterized by an imbalance between pro- and anti-inflammatory stimuli, and reflected in increased serum IgG levels. While, at 10 mg/kg, there is an activation of the immune system, with consequent recruitment of anti-inflammatory components, these are overtaken by more potent and/or abundant pro-inflammatory stimuli at 50 mg/kg. IgG levels are similar at 10 and 50 mg/kg because, although the concentrations of pro- and anti-inflammatory components may differ in both conditions, they vary proportionally. In turn, at 25 mg/kg, the intermediate dose, there might be an anti-inflammatory compensation of pro-inflammatory stimuli, explaining comparable IgG levels between the controls and this condition. Therefore, a combination of time- and dose-dependent effects is suggested to underlie seemingly inconsistent IgG results. The analysis of pro- and anti-inflammatory balance along the exposure time, instead of endpoint results, would add information on this hypothesis.

On the other hand, our results showed that tapentadol highest dose causes a decrease in IL-17A, a pro-inflammatory cytokine that acts in concert with TNF-α to induce the production of many other cytokines, chemokines and prostaglandins. In fact, alterations in inflammatory parameters were already reported as a tramadol effect, after treatment for 15 consecutive days (45 mg/kg during the first week and 90 mg/kg during the second week); serum interferon gamma (IFN-γ) decreased, while alterations in interleukin-10 (IL-10) serum levels were not detected [84]. Consistently with this, rats intraperitoneally injected with 1 mg/kg tramadol showed decreased IL-6 and unchanged interleukin-2 (IL-2) levels, although 10 and 20 mg/kg doses reversed alterations in IL-6 [85]. It was previously suggested that 5-HT reuptake inhibition could be involved in the immune effects of tramadol [85]. In accordance, histopathological examination of different tissues, such as lung, heart and brain, after H & E staining, showed that tramadol was associated with less inflammatory cell infiltrates than tapentadol [55]. In this sense, considering tramadol analgesic potency and lower immunosuppressive effects, it was suggested as a better alternative for pain treatment than classical opioids, since it may have an immune enhancing effect and, thus, be especially considered in conditions where immunosuppression is contraindicated [80,85,86,87].

Our study provides seemingly contradictory evidence on tramadol and tapentadol potential for inflammatory modulation—increased CRP and TNF-α for both opioids but decreased IL-17A and increased IgG for tapentadol, along with variable degrees of histological evidence of inflammation. Although it might be hypothesized that anti-inflammatory pathways are being recruited to compensate for inflammatory injury, it should be emphasized that more studies are needed to better understand tramadol and tapentadol role in the modulation of the immunological and inflammatory profiles.

### 3.3. Repeated Administration of Tramadol and Tapentadol Leads to Cardiac Muscle Cell Damage, though with No Impact on Ventricular Function

Electrocardiographic changes are one of the side effects associated with tramadol use, and clinical, hematological, and toxicological findings, such as troponin and myoglobin elevation, suggest myocardial damage upon intoxication with this opioid [48,88,89]. Cardiac troponin I was significantly elevated in rats receiving 12.5–300 mg/kg tramadol per day for two weeks [90]. Acute doses of tramadol and tapentadol were also shown to be cardiotoxic at the same doses used in the present study [55]. Thus, a series of serum biomarkers was assessed in order to study the putative impact of the repeated administration of tramadol and tapentadol on cardiac muscle cell integrity and function (Figure 2a). While serum BNP levels remained unchanged upon opioid treatment, CK-MB activity was found to increase upon exposure to tapentadol, while those of LDH and α-HBDH increased upon exposure to both opioids.

BNP is produced in cardiac ventricles, serving as a quantitative marker of heart failure; it proportionally reflects ventricular systolic and diastolic dysfunction, as well as acute hemodynamic change [91,92]. The lack of alterations in this biomarker suggests that tramadol and tapentadol do not impair these aspects of cardiac function.

Isoenzymes CK-MB, LDH_1–2_, and α-HBDH are found mainly in heart muscle, for which they are used as cardiac biomarkers [93], often with a good correlation with oxidative stress and inflammatory parameters [94]. α-HBDH is considered to represent LDH_1_ activity alone or both LDH_1_ and LDH_2_ activities [93]; consistently, in our study, the changes in LDH and α-HBDH were in the same direction and detected upon both tramadol and tapentadol treatment. Since LDH is an unspecific cell lysis biomarker, LDH_1-2_ also occur in rat kidneys in considerable amounts and nephrotoxicity has been reported under these experimental conditions [57], CK-MB arises as the most sensitive indicator of myocardial damage [93]. Accordingly, serum CK and CK-MB activities, as well as cardiac troponin I, were elevated in a case of multiple organ dysfunction after tramadol overdose [48].

Hence, the analysis of cardiac biomarkers as a whole is indicative of myocardial injury following repeated administration of both opioids.

### 3.4. Repeated Administration of Tramadol and Tapentadol Modifies Brain Cortex Metabolism, with Tapentadol Causing a Higher Degree of Metabolic Modulation

Aiming to investigate whether consecutive administration of therapeutic doses of tramadol and tapentadol impacts brain cortex metabolic profile, we have quantified metabolic parameters in the corresponding homogenates (Figure 2b).

The results are in agreement with those from previous studies by our own group, where we used the same animal model and opioid doses, but in an acute treatment context [55]. Although no significant changes were detected in serum glucose levels, brain cortex lactate contents were found to be elevated at the highest dose for both opioids. These changes were matched by an increase in the activity of LDH, the catalyst of lactate production, at all tapentadol doses. This is an extension of the effect observed in acute settings, where a significant elevation was limited to 50 mg/kg tapentadol [55]. We have previously shown that the exposure to tramadol and tapentadol affects the expression of energy metabolism enzymes [54], leading to a possible bioenergetic crisis that is supported by other studies [95,96,97] and reflected in lactate overproduction and decreased ATP synthesis. Interestingly, supratherapeutic tramadol doses have been found to partially inhibit the activities of respiratory chain complexes I, III, and IV, correlating with increased oxidative stress and explaining clinical and histopathological effects such as seizures and apoptosis [97]. Increased lactate levels were also observed in rat spinal cord dorsal horn upon acute and chronic morphine administration [98]. In parallel, astrocytic glycolysis and lactate production are closely associated with the astrocytic reuptake of glutamate and with neuronal oxidative metabolism, which is fueled by lactate [99]. This prompted us to further investigate the expression of astrocytic markers, namely GS, given lactate role in glutamine/glutamate cycling.

In turn, CK catalyzes the reversible phosphorylation of creatine to phosphocreatine, a highly diffusible energy carrier [100]. Brain CK is reported to locally fuel ATPases by providing phosphocreatine, as well as to maintain local ATP buffering under limited oxygenation and/or nutrient supply, where mitochondrial function and phosphocreatine regeneration is partially or totally impaired [100]. Increased expression of brain CK may therefore be regarded as an adaptation to opioid-induced stress and mitochondrial dysfunction.

Since LDH and CK activities were significantly augmented upon tapentadol exposure only, it might be deduced that this opioid causes greater brain metabolic modulation.

### 3.5. Repeated Exposure to Tramadol and Tapentadol Alters the Expression of Lung, Heart and Brain Toxicity Biomarkers at the Gene and Protein Levels, Correlating with Oxidative Stress, Inflammation, Metabolic and Histological Parameters

To ascertain the potential impact of tramadol and tapentadol repeated administration on gene and protein expression levels, a panel of toxicity biomarkers was assayed in lung, heart, and brain cortex samples from Wistar rats exposed to 50 mg/kg opioid, the highest dose under study. Alterations were found for most of these biomarkers (Figure 3), with their nature and extent being similar for most of the genes studied. We hypothesize the exceptions to be due to differences in tramadol and tapentadol structure, chemical properties and mechanisms of action. These account for different pharmacokinetics and pharmacodynamics and, consequently, for different potency and effects on target organs [14], which possibly include gene expression. Nevertheless, it should be underlined that changes in mRNA transcript levels do not necessarily translate into protein expression, since there might be posttranscriptional and posttranslational events affecting mRNA and protein stability.

Regarding the lung toxicity biomarker panel (Figure 3a), CC16 gene expression levels were found to be decreased upon both tramadol and tapentadol exposure. CC16, a protein with anti-inflammatory and immunomodulatory activities, is the major secretory product of the Clara cells, which play an important role in bronchial epithelial repair mechanisms [101,102]. Clara cells have the highest levels of CYP450 in the lung and are the main site of xenobiotic detoxification, rendering them particularly sensitive to injury, due to the production of toxic metabolites [101,103]. In this sense, tramadol bioactivation by CYP450 should not be overlooked, since there is evidence of pulmonary expression of isoenzymes such as CYP2B6 and CYP3A4 [104], supporting the possibility that tramadol metabolites may contribute to its pneumotoxicity. Clara cell destruction leads to decreased CC16 production, for which bronchoalveolar lavage or serum CC16 has been reported as a sensitive indicator of bronchial or epithelial injury. Accordingly, its decrease has been described in smokers and in occupational groups with an history of chronic exposure to several air pollutants [101,105], as well as in subjects with respiratory disease [102,103]. In mice models, reduced CC16 levels are associated with pulmonary inflammation and injury, alveolar septal cell apoptosis, airway mucus metaplasia, emphysema, and small airway remodeling [103,105]. Cigarette smoke-exposed *CC16^−^*^/*−*^ mice show increased lung levels of pro-inflammatory mediators chemokine (C-C motif) ligand 5 (CCL5) and matrix metalloproteinase-9 (MMP-9) and pro-fibrotic mediator transforming growth factor-β1 (TGF-β1), but lower levels of anti-inflammatory IL-10 than their wild-type counterparts [103]. Thus, pulmonary inflammation, dysfunction, and remodeling might be indicative of possible effects of repeated exposure to 50 mg/kg tramadol and tapentadol.

MCP-1 expression was also found to be downregulated upon exposure to both opioids. Although MCP-1 is a potent profibrotic chemokine, its plasma concentration has been found to be reduced in patients with a higher grade of pulmonary toxicity 1 h after radiotherapy [106,107], as well as upon exposure to some drugs and pollutants [108,109]. Since it has been implicated in alveolar tissue repair and, thus, in the resolution of inflammation, reduced MCP-1 expression might contribute, at least in part, to alveolar collapse and structural changes observed through histopathological analysis (Figure 5). Interestingly, in vitro TGF-β and TNF-α co-treatment decreased MCP-1 gene and protein expression in endothelial cells [110]. Since their levels have been found to be increased in our study—in heart tissue, at the gene level, for TGF-β (Figure 3b), and in serum samples, at the protein level, for TNF-α (Figure 2a), a similar correlation with MCP-1 under-expression might be hypothesized. Furthermore, heme oxygenase-1 (HO-1) induction, which we have previously shown to occur in the experimental conditions assayed in the present work [57], has been associated with a decrease in ROS and MCP-1 [111], thus providing an additional possible explanatory mechanism.

Matrix metalloproteinases are a family of endopeptidases involved in extracellular matrix (ECM) degradation and remodeling, being implicated in innate immunity, tissue repair, and homeostasis, but also in inflammation, modulation of bioactive compounds, apoptosis, and in the progression of several diseases, including xenobiotic-induced interstitial lung disease [112,113,114]. MMP-7, also known as matrilysin, is highly overexpressed in human idiopathic pulmonary fibrosis, participating in neutrophil transepithelial efflux and in fibrotic response [112,114,115]. In fact, it has been suggested as a reliable prognostic biomarker for lung disease [114,115]. Thus, MMP-7 overexpression upon treatment with 50 mg/kg tramadol might be correlated with the histopathological alterations observed for this condition (Figure 5), representing a possible predictor of lung function decline and disease progression.

In turn, besides being part of the first line of immune defense in the lung, surfactant proteins SP-A and SP-D, mainly secreted by type II pneumocytes and Clara cells, control inflammation and fibrosis and participate in the organization, stability, and metabolism of lung parenchyma [116,117]. Importantly, they contribute to the structural and functional integrity of pulmonary surfactant, thus avoiding alveolar collapse by reducing the surface tension at the air/liquid interface [118]. Their serum concentrations increase in pulmonary alveolar proteinosis, idiopathic pulmonary fibrosis, interstitial pneumonia with collagen vascular diseases, asthma, and respiratory distress syndrome [119,120], having been proposed as good differential diagnosis and prognosis biomarkers for idiopathic pulmonary fibrosis [117]. Interestingly, they were suggested to protect lungs from xenobiotic-induced oxidative injury, as deduced from correlations with TBARS levels in rat lung [116]. A similar correlation may be done in our study, considering the increase in lung TBARS contents upon treatment with both opioids (Figure 1a), SP-A gene overexpression upon tramadol treatment, and SP-A and SP-D gene overexpression upon tapentadol treatment. Therefore, gene expression results may reflect increased oxidative stress in opioid-treated rat lungs, as well as susceptibility to pulmonary disease.

IL-6 gene expression levels were quantified within the scope of the analysis of cardiotoxicity biomarker genes (Figure 3b), having increased upon tramadol treatment. IL-6 is a pleiotropic cytokine that connects innate and adaptive immunity and plays different roles throughout time. Although, in the short term, IL-6 initiates acute phase response and wound healing, directs immune cell activation and trafficking, having a pro-inflammatory and protective effect, it becomes pathogenic in a chronic context [121]. Chronically elevated IL-6 concentrations are associated with chronic inflammation, fibrotic disorders, myocardial hypertrophy, reduced contractility, remodeling and, ultimately, heart failure [121]. Increased IL-6 gene expression upon tramadol treatment might thus correlate with the observed higher degree of histopathological alterations, including inflammation and fibrosis (Figure 6). In addition, IL-6 abnormalities lead to dyslipidemia and cardiac lipotoxicity, although it is unclear whether excess or deficiency is responsible [122]. Given that serum lipid alterations were identified in the experimental conditions under study [57], such hypothesis should not be disregarded.

Plau/UPA is a serine protease that is suggested to play a role in cardiac fibrosis, since its absence seems to impair fibroblast ability to migrate into infarcted tissue, synthetize collagen and form fibrotic scars [123]. However, besides being active at sites of tissue remodeling and inflammation, Plau/UPA was paradoxically proven to protect heart tissue from oxidative damage, by promoting DNA repair [124]. Therefore, increased expression of Plau/UPA in cardiac tissue upon repeated administration of 50 mg/kg tramadol might simultaneously be associated with the histological evidence of fibrosis observed for this condition (Figure 7) and with an attempt to curtain opioid-induced oxidative damage. Interestingly, although we did not specifically measure DNA oxidation biomarkers, our results indicate that the heart tissue is the least affected by oxidative injury (Figure 1b), probably reflecting the activation of antioxidant defense mechanisms.

TGF-β superfamily members are central players in cell proliferation, differentiation and migration of different components of the cardiovascular system, being involved in cardiac hypertrophy, fibrosis, contractility, metabolism, angiogenesis, repair, remodeling, and regeneration [125,126]. Specifically, TGF-β2 is upregulated and undergoes de novo synthesis promptly after infarction and ischemic injury [125,126]. Several studies indicate that, in an infarcted myocardium, TGF-β family members regulate immune function by modulating chemotaxis, chemokine synthesis, immune cell differentiation and activation [126]. In addition, they have anti- or pro-apoptotic actions, enhance cardiomyocyte performance, promote myofibroblast conversion, stimulate ECM protein synthesis and have matrix-preserving effects, by inhibiting collagenase and increasing tissue inhibitor of metalloproteinase (TIMP) levels. Fibroblast activation or conversion into myofibroblasts drives ECM accumulation and pathological fibrosis [126]. Additionally, TGF-β stimulation was also shown to lead to endothelial-to-mesenchymal transition, which contributes to capillary rarefaction, tissue ischemia and consequent fibrotic myofibroblast deposition [127]. These roles are on the borderline between inflammation and repair, which might be a reasonable scenario upon treatment with both tramadol and tapentadol, considering that, in our study, TGF-β2 gene expression increased in both situations. Furthermore, upon tramadol exposure, TGF-β2 higher overexpression, combined with that of TIMP-1, is in line with the histological evidence of fibrosis and structural changes observed for this condition (Figure 7).

In turn, TIMPs maintain the homeostatic balance of myocardial ECM by inhibiting activated matrix metalloproteinases (MMPs) and their ECM-degrading function [128,129]. Elevated tissue and plasma TIMP-1 levels have been correlated with myocardial fibrosis and diastolic dysfunction, both in human patients and in animal models, through both MMP activation-dependent and -independent mechanisms [128,129]. Thus, there might be an association between TIMP-1 gene overexpression and the evidence of cardiac fibrosis observed at 50 mg/kg tramadol (Figure 7). Moreover, TIMP-1 gene overexpression in heart tissue, following tramadol repeated administration, may be correlated with that of IL-6, since this pro-inflammatory cytokine may directly upregulate TIMP-1 expression, suggesting common regulatory pathways [130].

Neurotoxicity biomarkers have been quantified at the gene (at the highest opioid dose) and protein (at all doses tested) levels (Figure 3c and Figure 4, respectively). As previously noted, though a correlation is expected, changes in mRNA levels might not be reflected in protein expression, due to posttranscriptional and posttranslational effects modulating mRNA and protein stability.

α-Synuclein, one of the biomarkers assayed, is associated with synaptic vesicular trafficking, transmission, and plasticity [131,132]. Aggregates of misfolded, toxic forms are reported in synucleinopathies, having been associated with alterations in structural cell components, multiple cellular pathways, protein clearance mechanisms and mitochondrial function [131]. This pre-synaptic protein has also been shown to negatively regulate dopaminergic neurotransmission, since it decreases the expression and inhibits the activity of enzymes involved in dopamine synthesis, affects the activity of dopamine transporters and the capacity of refilling and storage of pre-synaptic dopamine-containing vesicles [132,133]. Such observations are highly suggestive of α-synuclein participation in opioid-elicited effects on the dopaminergic reward pathway [132]. Indeed, besides leading to cellular stress and toxicity, increases in α-synuclein levels have been associated with predisposition to addiction to different drugs of abuse, such as cocaine and alcohol [132,133,134]. Although α-synuclein mRNA levels decreased upon exposure to 50 mg/kg tramadol and tapentadol (Figure 3c), such alterations were reflected at the protein level for 50 mg/kg tramadol, 10 and 25 mg/kg tapentadol only (Figure 4). In fact, its protein levels increased at 25 mg/kg tramadol and were unchanged at 50 mg/kg tapentadol (Figure 4). The same trend was observed in mice brains upon chronic morphine treatment and 48 h of withdrawal; while downregulation of α-synuclein mRNA was observed in the basolateral amygdala, dorsal striatum, nucleus accumbens, and ventral tegmental area, its protein levels were significantly increased in the amygdala and striatum/accumbens. The authors of the study argue that opposite changes in gene and protein levels might take place in different populations of projection neurons whose somata locate in distinct brain areas [132]. In addition, posttranslational mechanisms, such as phosphorylation and ubiquitination, influence α-synuclein degradation rate and stability, further possibly explaining differences between mRNA and protein levels [132]. Other opioid exposure-studies reiterate such inconsistencies, as α-synuclein protein levels decrease in human serum [133], but increase in brain paranigral nucleus and substantia nigra ventral part after chronic heroin use [134]. In turn, α-synuclein protein levels increase in neuroblastoma cells after chronic exposure to morphine [135], as well as in rat forebrain cortex upon a 10-day exposure to the same drug [136], but decrease in rat hippocampus under the same conditions [137]. Therefore, a combination of brain area-specific phenomena and posttranslational mechanisms regulating protein stability might account for discrepancies between α-synuclein gene and protein expression levels.

BDNF is a small neurotrophin that is also involved in pain transmission, neuroinflammation, neuromodulation, memory, learning, addiction behavior, and opioid analgesic tolerance [138,139,140]. Upregulation of BDNF and its receptor has been suggested as an important neuroadaptation, being implicated in synaptic plasticity and neuronal survival [139]. Indeed, BDNF gene and protein overexpression has been reported in lumbar spinal cord samples from morphine-tolerant mice [138] and in hippocampal samples from rats repeatedly administered with the same opioid, but not upon acute exposure [141]. Increased BDNF serum concentrations were also reported for heroin-dependent male patients undergoing methadone maintenance treatment [140]. However, in line with our results, a decrease in BDNF-encoding mRNA levels was detected in Wistar rat brain cortical areas upon repeated daily i.p. injection of 20 mg/kg tramadol for 21 days, while no changes were identified in hippocampus, both in short- and long-term contexts [139,142]. The authors theorize that, unlike antidepressant drugs, for which neurotrophic effects have been postulated, tramadol does not induce such kind of neuroadaptation [139]. Likewise, acute and repeated i.p. injections of 1–10 mg/kg tapentadol to rats did not lead to changes in BDNF transcript levels in ganglia and central tissues [143]. The influence on BDNF levels might thus depend on the opioid used, on the brain region under analysis, and on the exposure regimen.

Opioid brain signaling and information processing were found to induce the activation of glial cells, especially astrocytes, by direct MOR stimulation in astrocyte membranes [144,145]. GS is highly expressed in astrocytes, for which it serves as an astrocytic marker [146]. It is a key regulatory enzyme in brain glutamate and glutamine dynamics, which, in turn, is involved in opioid addiction and dependence. Tight control of glutamate extracellular levels is crucial, not only for nociception neurotransmission, but also to avoid neuronal over-excitation and excitotoxicity [147]. Glucose taken by astrocytes is metabolized via glycolysis into lactate, thereby producing ATP to meet energy requirements, mostly for glutamate reuptake from the synaptic cleft [99,146]. Glutamate may also be synthetized from α-ketoglutarate, a Krebs cycle intermediate, through transamination via mitochondrial aspartate aminotransferase. Glutamate is then condensed with toxic ammonia, via GS, to form non-toxic glutamine [99,146]. This, in turn, is transported to presynaptic terminals, where it is converted into glutamate in excitatory synapses and γ-aminobutyric acid (GABA) in inhibitory synapses [99,146]. Astrocytic glucose consumption and lactate production appear to be largely coupled by the astrocytic reuptake of glutamate released at excitatory synapses, with the lactate produced by astrocytic glycolysis serving as a substrate for neuronal oxidative metabolism [99]. Studies on GS expression yield contradictory results. While some proteomic analyses report a decrease in GS levels following morphine administration, others—including one study with rat cerebral cortex synaptosomes—report an increase [148]. When GS activity was measured instead of its protein levels, no changes were found [148]. Muscoli and co-authors added that, although GS total protein levels did not change after morphine repeated administration, the levels of its nitrated, inactivated form increased, which might represent a contributory mechanism for antinociceptive tolerance [149]. In turn, GS activity increased in different brain regions upon Wistar rat daily injection with 31 mg/kg tramadol for 3 consecutive days, peaking on day 3 or 6 post-administration [150]. Opioid peptides also led to an increase in GS activity in cell lines with astrocytic phenotype [151]. The authors conclude that astrocytes respond to opioids and argue that GS increased activity contributes to brain glutamate mobilization and compartmentation and, consequently, to prevent its pathological effects [150,151,152]. Such argument may explain our own results, in view of GS increased expression at low and intermediate opioid doses (as determined through Western blotting, Figure 4) and at the highest opioid dose (as determined through quantitative Real-Time PCR (qRT-PCR), Figure 3c). Furthermore, we found lactate and LDH levels to be increased in brain cortex homogenates (Figure 2b), which might be correlated with our GS gene expression results, in view of the interdependence between lactate and glutamate metabolism.

S100β, another astrocytic marker, plays a key role in neuroinflammation by activating signaling cascades that lead to the production and secretion of inflammatory cytokines. Its levels increase in hippocampal tissue, cerebrospinal fluid and serum during anoxic brain damage and pathophysiological situations, acting as a neuroapoptotic factor [153,154]. Serum S100β levels increased in pediatric patients following general anesthesia by a combination of fentanyl with non-opioid drugs; only the total dose of fentanyl was significantly correlated with the difference between post-exposure and baseline S100β levels [154]. In turn, acute administration of remifentanil also led to increased serum S100β levels in rats, which was associated with cognitive dysfunction [155]. However, Kuklin and co-authors found no changes in S100β serum levels between control and morphine-treated Wistar rats subsequently subjected to asphyxia cardiac arrest [156]. In line with this study, we have found no statistically significant differences between control and opioid-exposed groups, as far as S100β gene expression is concerned (Figure 3c). We hypothesize that the extent of brain injury, as assessed through this biomarker, is lower than that caused by other opioids.

In the present study, the protein content of GFAP, another astrocyte activation biomarker, was also found to be increased in brain cortex extracts upon exposure to the lowest and intermediate opioid doses (Figure 4). GFAP hippocampal immunoreactivity increased in juvenile and adult mice treated with 40 mg tramadol/kg/day for 1 month, which, along with astrocytic swelling, was reported as astrogliosis [144]. Morphine exposure has been reported to lead to similar effects in different brain areas, including ventral tegmental area, nucleus accumbens, striatum, and frontal cortex [145,157,158]. Several studies support the role of GFAP upregulation in opioid dependence and tolerance [145,157,158]. Indeed, chronic drug abuse-induced astrogliosis is considered an innate immunity response to neurotoxicity and brain damage, which may lead to alterations in synaptogenesis and neurogenesis, apoptosis and/or necrosis [144,158]. Thus, in our study, increased GFAP expression is compatible with the signs of glial proliferation and hypertrophy observed in histopathological examination; these, in turn, are a response to opioid-induced injury (Figure 8). Given the roles of GS and GFAP, their increased protein expression for the lowest opioid doses might be hypothesized as an attempt to reduce neurotoxicological injury, which is lost at the highest opioid doses, due to damage accumulation.

Interestingly, there seems to be a dissociation between glial and metabolic markers, since, while tramadol induces glial alterations, as determined through qRT-PCR, Western blotting, and histological analysis (Figure 3c, Figure 4 and Figure 8 respectively), it does not appear to significantly affect brain metabolism, apart from its effect on lactate concentrations (Figure 2b).

### 3.6. Repeated Exposure to Tramadol and Tapentadol Leads to Histopathological Damage in Lung, Heart and Brain Cortex Tissues from the Lowest Therapeutic Dose

In addition to analyzing the effects of a repeated exposure to clinical doses of tramadol and tapentadol at the molecular, biochemical, and metabolic levels, we have also studied their impact on lung, cardiac, and brain cortex histopathology. In fact, several histopathological alterations were documented by our own group in liver and kidney, following acute and repeated administration of the therapeutic doses of tramadol and tapentadol used in the current study [56,57], whilst lung, heart, and brain cortex tissue alterations had been described in an acute context [55].

Pulmonary fibrosis, congestion, edema, emphysema, and endoalveolar hemorrhage are reported as autopsy findings in fatal poisonings by tramadol or M1, alone or in combination with other drugs [36,39,40,43,44,45,46,48,88,159,160,161], as well as by tapentadol [33]. Furthermore, lung histopathological alterations were reported in animal models following acute and chronic administration of therapeutic and supratherapeutic doses of both opioids [55,58,59]. Interstitial alterations comprise pulmonary congestion, hemorrhage, fibrin deposition, inflammatory infiltrates, edema and fibrosis, while alveolar changes include alveolar wall and septa thickening and destruction (emphysema) to varying extents, as well as intra-alveolar edema and hemorrhage [58,59]. In our previous acute administration assays, interstitial congestion and hemorrhage were dose-dependent for tramadol, while they were observed at all tapentadol doses; in turn, alveolar collapse became evident at the highest doses [55]. The results of the present study point predominantly to alveolar alterations—alveolar wall thickening and collapse, cellular hyperpigmentation and disorganization, which are now dose-independent. Such findings are compatible with CC16, MCP-1 and MMP-7 gene expression results (Figure 3a). It is noteworthy that alveolar destruction and loss of parenchyma are more evident for tapentadol, corroborating our previous postulate that this opioid causes lung damage to a greater extent [55].

With respect to cardiac tissue, our study has evidenced altered cardiomyocytes, fiber filament disorganization and heterogeneous pigmentation, loss of striation, inflammatory infiltrates, and fibrous tissue deposition both through H & E and Masson’s trichrome staining methods (Figure 6 and Figure 7, respectively). Such results are in line with those from our previous acute exposure studies [55]; however, in contrast to these, histopathological findings are now evident even at the lowest dose for tramadol and are more profuse for both opioids. In particular, while fibrous tissue deposition was not even suspected upon single exposure, it is now supported by both staining methods, as well as by the gene expression changes of the cardiotoxicity biomarkers assayed (Figure 3b). The signs of fibrosis are more evident after tramadol treatment, which also led to more intense alterations in the expression of cardiac markers. The origin of myocardial fibrosis in opiate use is still unclear [162]. Nevertheless, similarly to our results, heroin users have been reported to present up to a 5-fold increase in the number of inflammatory cells in the myocardium, suggesting a general activation of the cellular immune system and pointing to post-inflammatory focal interstitial fibrosis [161,162]. Hypereosinophilic bundles, congestion, hemorrhage, and leukocytic infiltration were also reported in cardiac tissue from rabbits submitted to short- and long-term passive opium smoking; minimum evidence of myonecrosis was reported for long-term exposure only [163]. Regarding postmortem investigation evidence, cardiomegaly is declared in autopsy reports of tramadol and tapentadol fatal poisoning cases [33,39,40,159].

In relation to brain cortex histological analysis, the continuous exposure to clinical doses of tramadol and tapentadol led mainly to neuron swelling and degeneration. Though they are observed in all conditions, these alterations accumulate along with tapentadol dose, whereas they are more profuse and diverse at all tramadol doses; neurons with irregular morphology are also more evident upon exposure to this opioid. Glial and microglial cells are now observable at all doses, consistently with the increase in astrocytic markers GS and GFAP (Figure 3c and Figure 4), while they were more evident for intermediate and highest doses in single-exposure assays [55]. Such observations strengthen our previous hypothesis that tapentadol might not be so comparatively advantageous in the treatment of neuropathic pain, despite having a lower inhibitory effect on hippocampal neurogenesis [27]. These results are also in line with those from similar studies, mostly concerning rat consecutive administration with tramadol doses ranging from 25 to 200 mg/kg, for periods up to 60 days. Such studies report disorganized cortical layers and hypercellularity [59,60,61,62], as well as degenerated, vacuolated neurons, irregular in shape, with pyknotic and vacuolated nuclei, often with heterogeneous pigmentation and evident signs of apoptosis [58,59,60,61,62,70,164,165,166]. Neuronal degeneration and histological changes have been correlated with glucose metabolism alterations and with the bioenergetic crisis discussed in Section 3.4 [55,60]. Cellular infiltrates are also mentioned in these studies [58,60,164], as well as gliosis, satellitosis, and microglial and oligodendrocyte proliferation [58,62,144,164]. Vascular dilatation and congestion, hemorrhage, and brain edema are also cited [58,59,60,61,62,70,164,166]. Indeed, severe brain edema and hypoxic brain damage are reported in opiate-related deaths, including those from tramadol [39,40,45,161].

**Figure 9:**
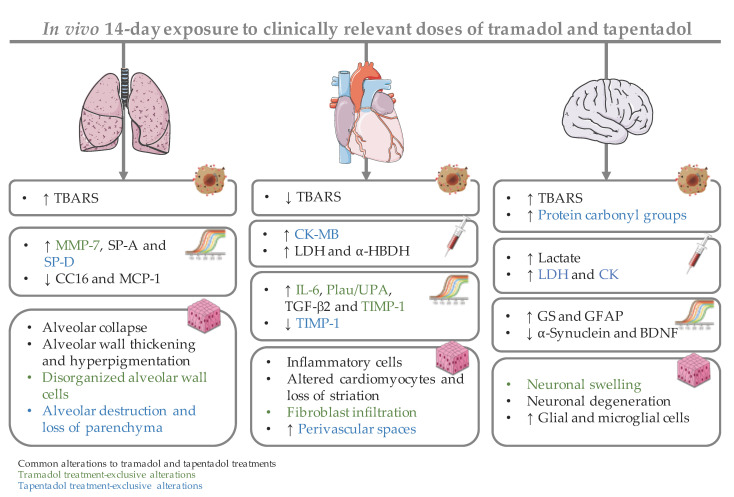
Schematic representation of the pulmonary, cardiac, and brain cortex effects of a 14-day exposure of Wistar rats to clinically relevant doses of tramadol or tapentadol, assessed at the molecular, oxidative stress, metabolic and histological levels. α-HBDH: α-hydroxybutyrate dehydrogenase; BDNF: brain-derived neurotrophic factor; CC16: Clara cell protein-16; CK-MB: creatine kinase muscle brain isoform; CK: creatine kinase; GFAP: glial fibrillary acidic protein; GS: glutamine synthetase; IL-6: interleukin-6; LDH: lactate dehydrogenase; MCP-1: monocyte chemoattractant protein-1; MMP-7: matrix metalloproteinase-7; Plau/UPA: plasminogen activator, urokinase; SP-A: pulmonary surfactant protein A; SP-D: pulmonary surfactant protein D; TBARS: thiobarbituric acid reactive substances; TGF-β2: transforming growth factor-β2; TIMP-1: tissue inhibitor of metalloproteinase-1.

Collectively, our results show that lung, heart, and brain cortex toxicological damage occurs at the biochemical, metabolic, and histological levels upon exposure to clinical doses of tramadol and tapentadol (Figure 9).

**Figure 10:**
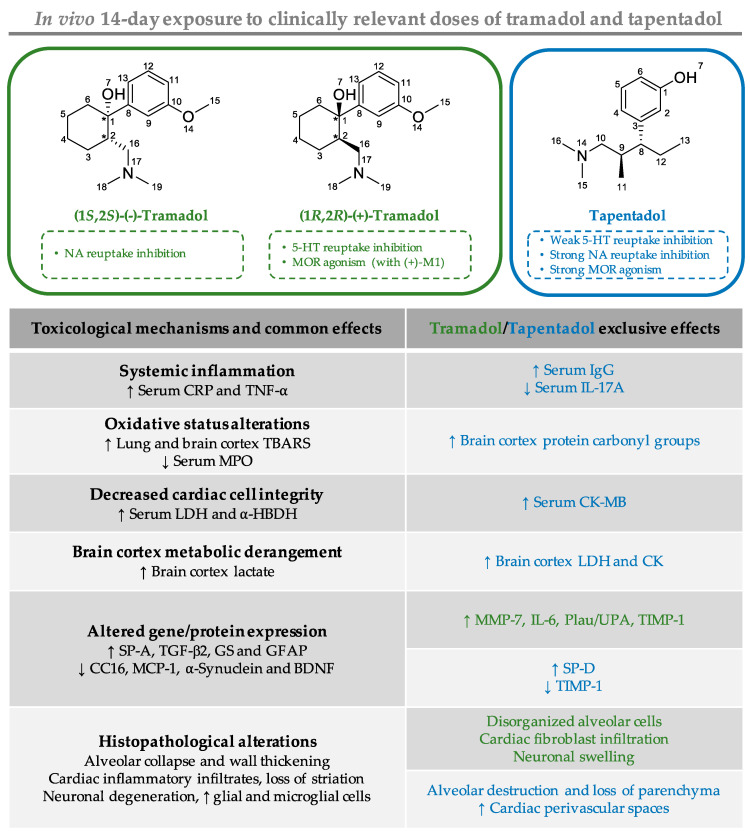
Summary of the toxicological mechanisms associated with a 14-day exposure of Wistar rats to clinically relevant doses of tramadol or tapentadol. 5-HT: serotonin; α-HBDH: α-hydroxybutyrate dehydrogenase; BDNF: brain-derived neurotrophic factor; CC16: Clara cell protein-16; CK-MB: creatine kinase muscle brain isoform; CK: creatine kinase; CRP: C reactive protein; GFAP: glial fibrillary acidic protein; GS: glutamine synthetase; IgG: immunoglobulin G; IL-17A: interleukin-17A; IL-6: interleukin-6; LDH: lactate dehydrogenase; M1: *O*-desmethyltramadol; MCP-1: monocyte chemoattractant protein-1; MMP-7: matrix metalloproteinase-7; MOR: μ-opioid receptor; MPO: myeloperoxidase; NA: noradrenaline; Plau/UPA: plasminogen activator, urokinase; SP-A: pulmonary surfactant protein A; SP-D: pulmonary surfactant protein D; TBARS: thiobarbituric acid reactive substances; TGF-β2: transforming growth factor-β2; TIMP-1: tissue inhibitor of metalloproteinase-1; TNF-α: tumor necrosis factor-α.

As seen in our study addressing hepatorenal toxicity following consecutive opioid administration [57], lower therapeutic doses are able to induce injury if administered repeatedly. Damage accumulates along lengthier exposure periods than those we have previously assayed [55,56], but shorter than those employed in most peer studies. Figure 10 summarizes tramadol and tapentadol mechanisms of action, as well as the common and exclusive toxicological effects found in our study. Overall, tapentadol appears to induce alterations in more oxidative stress, cardiac, and brain cortex metabolism biomarkers, while tramadol seems to have more histopathological impact (Figure 9 and Figure 10).

## 4. Materials and Methods

### 4.1. Chemicals

Tramadol and tapentadol hydrochloride salts were obtained from Sigma-Aldrich (St. Louis, MO, USA) and Deltaclon (Madrid, Spain), respectively, having been dissolved and diluted in saline (0.9 g/L (*w*/*v*) NaCl) for administration. Sodium thiopental was supplied by B. Braun Medical (Queluz de Baixo, Portugal). All other chemicals were commercial preparations of the highest available degree of purity.

### 4.2. Experimental Models and Animal Handling

In this experimental study, 42 male Wistar rats, aged 8 weeks and weighing 250–300 g, were provided by the i3S animal facility (Porto, Portugal). Animals were housed in acrylic cages, in an environment enriched with wood chips and paper towels, and maintained under controlled conditions (22 ± 2 °C, 50–60% humidity, 12/12 h light/dark cycles). They were given unlimited access to tap water and rat chow (standard short and middle period maintenance formula for rodents, reference 4RF21, Mucedola/Ultragene (Milan, Italy)), and kept under a quarantine period of at least one week before experimental assays.

Animal experimentation was conducted in conformity with the European Council Directive (2010/63/EU) guidelines, transposed into the Portuguese law (Decree-Law no. 113/7 August 2013). Experimentation approval was also obtained from the Ethics Committee of CESPU, Institute of Research and Advanced Training in Health Sciences and Technologies (IINFACTS), Gandra, PRD, Portugal (processes no. PI4AC 2017, PI4AC 2018 and PI-3RL 2019), and complied with the National Ethics Council for the Life Sciences (CNECV) guidelines.

### 4.3. Experimental Design and Drug Treatment

Following acclimatization, rats were randomized into 7 groups of 6 animals each. The sample size and number of animals per group were established through the G*Power software, version 3.1.9.6 (Heinrich-Heine-Universität Düsseldorf, Düsseldorf, Germany), assuming a significance level of 0.05, an 80% power and effect size values adjusted in accordance with the biochemical parameters under analysis, based on literature and on the previous experience of the team.

Drugs were delivered daily, via single 1 mL-i.p. injections, using saline solution (0.9% (*w*/*v*) NaCl) as vehicle. Administrations were conducted at the same time each day, throughout 14 consecutive days. Each group was injected with a specific dose of each opioid—10, 25 or 50 mg/kg tramadol or tapentadol, whereas the control group received saline solution administrations.

Human therapeutic doses were converted into the animal equivalent doses (AED) by assuming a body surface area correction factor (K*_m_*) of 6.2 and the following formula, for a 60 kg-human: AED (mg/kg) = Human dose (mg/kg) × K*_m_* ratio [167,168,169]. In line with that described in our previous studies, 10 mg/kg is equivalent to an effective, analgesic dose, whilst 25 and 50 mg/kg are equivalent to an intermediate and the maximum recommended daily dose, respectively [55,56,57].

Immediately upon the last administration, rats were transferred to metabolic cages and allowed free access to tap water, but no food, for the remaining 24 h. Animals were monitored along this period, and then they were sacrificed through anesthetic procedures (i.p. injection with 60 mg/kg sodium thiopental, dissolved in saline solution).

### 4.4. Collection and Processing of Biological Samples

Blood samples were collected with a hypodermic heparinized needle, through cardiac puncture. Serum was obtained through centrifugation at 3000× *g*, 4 °C, for 10 min. Samples were aliquoted and stored (−80 °C) for biochemical analysis.

Lungs, heart and brain cortex were surgically removed from each animal, dried with gauze and weighed on an analytical balance. A portion of each organ was homogenized in an Ultra-Turrax^®^ (IKA^®^, Staufen, Germany), in 1:4 (*w*/*v*) ice-cold 50 mM phosphate buffer (KH_2_PO_4_ + Na_2_HPO_4_ H_2_O), pH 7.4. Homogenates were submitted to centrifugation at 4000× *g*, 4 °C, for 10 min. Supernatants were aliquoted and stored at −80 °C, along with the remaining intact portions of the organs.

#### 4.4.1. Quantification of Oxidative Stress Parameters

Oxidative stress was assessed for LPO and protein oxidation, in lung, heart and brain cortex homogenates. TBARS and protein carbonyl groups (ketones and aldehydes) were used as LPO and protein oxidation biomarkers, respectively. Results were normalized against total protein content, which was determined through the Pierce™ BCA Protein Assay Kit (Thermo Scientific, Rockford, IL, USA), according to the manufacturer’s microplate procedure, and using 10-fold diluted homogenates.

Perchloric acid was added to each homogenate to a final concentration of 5% (*w*/*v*), to precipitate proteins. Acidified samples were centrifuged at 13,000× *g*, 4 °C, for 10 min; pellets and supernatants were stored at −80 °C. LPO quantification was performed in the supernatants, according to Buege et al. [170]. Results were expressed as nanomoles of MDA equivalents per milligram of protein. In turn, protein pellets were used for carbonyl group quantification, following the method reported by Levine et al. [171]. Results were expressed as 2,4-dinitrophenylhydrazine (DNPH) nanomoles incorporated per milligram of protein.

MPO activity was assayed in undiluted serum samples with the MPO Colorimetric Activity Assay Kit (Sigma-Aldrich), following the manufacturer’s recommendations. Results were expressed in terms of mU/mL.

In turn, the total antioxidant capacity was determined in undiluted serum samples, through the Total Antioxidant Capacity Assay Kit (Sigma-Aldrich), according to the manufacturer’s directions. Results were expressed in terms of mM of antioxidants (Trolox equivalents).

#### 4.4.2. Quantification of Biochemical/Immunological Parameters in Serum Samples and in Brain Cortex Homogenates

CRP, CK-MB isoform, glucose, α-HBDH and IgG were quantified in undiluted serum samples, while lactate and CK were determined in undiluted brain cortex homogenates. In turn, LDH activity was quantified both in serum and brain cortex homogenates. Biochemical/immunological analytes were quantified in an automated analyzer (Prestige 24i, Tokyo Boeki, Tokyo, Japan), following the manufacturer’s instructions, as previously reported [54,55,56,57,172], and using undiluted samples. Calibration was conducted for each parameter, by using two appropriate calibrators and plotting 5-point standard curves. Quality controls were also included. All automated analyzer reagents were supplied by Cormay PZ (Warsaw, Poland).

CK, CK-MB, α-HBDH, and LDH enzyme activities were determined as U/L. In turn, biochemical/immunological parameters were retrieved as mg/dL, except for CRP (mg/L). Results from homogenate determinations were further normalized against total protein content and are thus expressed as mg/dL/mg protein (lactate) or U/L/mg protein (CK and LDH).

TNF-α and IL-17A were determined in serum samples, through enzyme-linked immunosorbent assay (ELISA), using the ELISA MAX^TM^ Deluxe Set Rat TNF-α and ELISA MAX^TM^ Deluxe Set Rat IL-17A (BioLegend, San Diego, CA, USA), respectively. BNP was also determined, through enzyme immunoassay (EIA), in serum samples, using the Brain Natriuretic Peptide EIA Kit (Sigma-Aldrich). All determinations were performed in conformity with the manufacturers’ specifications. For IL-17A and BNP quantification, samples were diluted 2-fold with assay diluent, whereas undiluted samples were used for TNF-α analysis. Immunoassay results were expressed as pg/mL.

#### 4.4.3. Gene Expression Analysis Through qRT-PCR

Total RNA was extracted from lung, heart, and brain cortex samples using the NZYol reagent (NZYTech, Lisbon, Portugal), following the manufacturer’s instructions for tissues. RNA integrity was confirmed through 1.4% (*w*/*v*) agarose gel electrophoresis, while its degree of protein and organic compound contamination was determined as the optical density (OD) OD_260 nm_/OD_280 nm_ and OD_260 nm_/OD_230 nm_ ratios, respectively (NanoDrop 2000 spectrophotometer, Thermo Scientific). Samples presenting OD_260 nm_/OD_280 nm_ and OD_260 nm_/OD_230 nm_ ratios ≥ 1.8 were used for complementary DNA (cDNA) synthesis, which was performed from 800 ng total RNA, using the NZY First Strand cDNA Synthesis kit (NZYTech), according to the manufacturer’s directions.

Gene expression was analyzed using the iQ™ SYBR^®^ Green Supermix (Bio-Rad Laboratories, Hercules, CA, USA), following the supplier’s instructions. Each cDNA sample was diluted 10-fold in ultrapure water and analyzed in duplicate, thereby totaling 12 replicates for each experimental condition. CC16, MCP-1, MMP-7, SP-A, SP-D, IL-6, Plau/UPA, TGF-β2, TIMP-1, α-synuclein (SNCA), GS, BDNF, and S100β genes were analyzed. 18S ribosomal RNA (18S rRNA) housekeeping gene was used as a loading control.

Each amplification mixture was composed of 12.5 µL 2× iQ™ SYBR^®^ Green Supermix (Bio-Rad Laboratories), 2 µL diluted cDNA (equivalent to 8 ng cDNA), forward and reverse primers (STABvida, Caparica, Portugal) to a final concentration of 100 nM each, and 10 µL RNase-free water, thus totaling a final volume of 25 µL. Primer sequences are specified in Table 1. RNA template controls (RTC) and non-template controls (NTC) were included in each run.

The qRT-PCR program was run in a C1000™ Thermal Cycler, equipped with a CFX96™ Real-Time System (Bio-Rad Laboratories). It comprised an initial denaturation step at 95.0 °C for 3 min, 35–41 amplification cycles composed of a denaturation step at 94.0 °C for 20 s, an annealing step for 30 s, an extension step at 72.0 °C for 30 s and a plate read step. The number of amplification cycles and the annealing temperatures used in the analysis of each gene are listed in Table 1. A melt curve was then acquired between 65.0 °C and 95.0 °C, with 0.5 °C increments at every 5 s, followed by plate reads.

Results were analyzed with the Bio-Rad CFX Manager software, version 3.1 (Bio-Rad Laboratories), and normalized against those of the control group. Relative changes in gene expression were quantified using the Δ(ΔCt) algorithm.

#### 4.4.4. Brain Cortex Protein Expression Analysis through Western Blotting

Brain cortex α-synuclein, GS and GFAP expression was also assessed at the protein level, by means of Western blotting assays. Brain cortex samples from each animal were homogenized in 1:5 (*w*/*v*) ice-cold RIPA lysis buffer (50 mM Tris HCl, pH 7.4, 150 mM NaCl, 5 mM EGTA, 1% (*v*/*v*) Triton X-100, 0.5% (*w*/*v*) sodium deoxycholate, 0.1% (*w*/*v*) SDS), supplemented at 1:100 with protease inhibitor cocktail (104 mM AEBSF, 80 μM aprotinin, 4 mM bestatin, 1.4 mM E-64, 2 mM leupeptin, 1.5 mM pepstatin A; Sigma-Aldrich), according to the supplier’s instructions. Lysates were incubated for 15 min on ice, and then centrifuged (15,000× *g*, 15 min, 4 °C) to remove cell debris. Supernatants were stored at −80 °C until further use. Total protein content was determined through the Pierce™ BCA Protein Assay Kit (Thermo Scientific), according to the manufacturer’s microplate procedure, and using 10-fold diluted protein extracts. 20 μg protein were loaded and separated by sodium dodecyl sulphate polyacrylamide gel electrophoresis (SDS-PAGE)—12% (GFAP and GS) or 15% (α-synuclein)—and transferred to a nitrocellulose membrane (110 mA, 75 min). Membranes were stained with Ponceau S to confirm sample transfer. They were then blocked with 5% (*w*/*v*) non-fat dry milk in TBST (20 mM Tris/HCl, pH 7.5, 150 mM NaCl, 0.1% (*v*/*v*) Tween 20) and probed with anti-GFAP mouse antibody (G-A-5) (1:2000, Merck Millipore, Burlington, MA, USA), anti-GS mouse antibody, clone GS-6 (1:500, Merck Millipore) or anti-α-synuclein mouse antibody (4D6) (1:500, Abcam, Cambridge, UK), diluted in 1% (*w*/*v*) non-fat dry milk in TBST, overnight at 4 °C. Then, membranes were incubated for 1 h, at room temperature, with appropriate horseradish peroxidase-conjugated secondary antibodies (Sigma-Aldrich), diluted 1:1500 in 1% (*w*/*v*) non-fat dry milk in TBST. To confirm equal protein loading, membranes were reprobed with anti-α-tubulin rabbit antibody (1:200, Abcam). Bands were visualized by treating the immunoblots through the enhanced chemiluminescence (ECL) method (Thermo Scientific) and scanned in a Gel Doc™ XR densitometer (Bio-Rad Laboratories). Densitometric analysis was performed with The Discovery Series™ Quantity One^®^ 1-D analysis software, version 4.6.5 (Bio-Rad Laboratories). Band intensities were normalized against those from α-tubulin and then against the control.

#### 4.4.5. Lung, Heart, and Brain Cortex Histopathological Analysis

One portion of lung, heart, and brain cortex tissue from each animal was fixed in 4% (*w*/*v*) formaldehyde for 24 h at room temperature, regarding histological analysis. It then underwent routine dehydration and paraffin wax-embedding procedures, as previously reported [186,187]. Three μm-thick sections were obtained in a Shandon™ Finesse™ 325 microtome (Thermo Scientific) and adhered to glass slides. H & E and Masson’s trichrome staining procedures were performed with heart samples, while lung and brain cortex samples were processed for H & E staining only. Slides were analyzed under phase contrast microscopy, in an Eclipse TE2000-U microscope (Nikon, Melville, NY, USA), coupled to a DXM1200F digital camera and controlled by the ACT-1 software, version 2.70 (Nikon). Multiple microscope fields of observation were analyzed, and the most representative ones were photographed using 100× and 600× magnifications.

### 4.5. Statistical Analysis

Statistical analysis was performed by Analysis of Variance (ANOVA), followed by Dunnett’s multiple comparisons test as post-hoc analysis. Data are presented as means ± SD and probability values of *p* < 0.05 were considered as statistically significant. Graphic plotting and all statistical tests were performed using GraphPad Prism^®^ version 8.3.1 (GraphPad Software, LLC, San Diego, CA, USA). In all quantifications, results were compared with those from the control animals, injected with saline solution.

## 5. Conclusions

Opioid abuse and misuse are a current trend and a worldwide concern. In spite of being designed to circumvent the mechanistic, pharmacokinetic and pharmacodynamic flaws of their predecessors, synthetic prescription opioids such as tramadol and tapentadol imply some degree of toxicological risk, especially if misused or used for prolonged periods. The study hereby reported successfully attempted to explore the molecular, metabolic and cellular mechanisms underlying the toxicological effects from a repeated exposure to two common prescription opioids. The repeated administration of therapeutic doses, instead of supratherapeutic ones or overdoses, enables an approximation to their real consumption conditions, often in clinical settings and on a subacute to chronic basis.

Our results evidence that the repeated exposure to tramadol and tapentadol clinically relevant doses elicits lung and brain cortex lipid peroxidation, as seen through increased tissue TBARS levels, along with a generalized inflammatory status, as deduced from augmented serum CRP and TNF-α. The results are also compatible with damage to cardiac tissue integrity, in view of elevated CK-MB, LDH, and α-HBDH activities, though such alterations are not reflected in ventricular dysfunction, as no changes were detected in serum BNP levels. In turn, consistently with previous studies, the brain cortex seems to undergo a shift towards anaerobic metabolism, given the increase in tissue lactate contents and in LDH and CK activities, which might be associated with neuronal degeneration. Histopathological evidence comprises findings as diverse as alveolar collapse and destruction, cardiomyocyte and cardiac fiber alterations, inflammatory infiltrates, fibrous tissue deposition between cardiomyocytes, neuronal degeneration, and glial and microglial cell accumulation. Changes in the expression levels of toxicity biomarker genes and proteins correlate well with the alterations detected in oxidative stress, inflammation, metabolic and histopathological parameters in all tissues under analysis.

The present study provides additional insights to our previous single-exposure assays focusing on acute liver, kidney, heart, lung, and brain cortex toxicity caused by the same opioid doses. Furthermore, it demonstrates that, instead of being restricted to metabolizing organs, the toxicological effects deriving from a repeated exposure do also occur in target tissues. Indeed, this is, to the best of our knowledge, the first study comparatively focusing on lung, cardiac and brain cortex toxicity following consecutive administration of tramadol and tapentadol. Our results not only add information to the interpretation of adverse events, but should also draw the attention of the scientific and medical communities to the need to carefully prescribe and use tramadol and tapentadol, particularly in the presence of cardiopulmonary and/or neuropathic concomitant disease, and/or if lengthy usage periods are required.

Although tapentadol is considered an upgrade over tramadol, in view of its more linear pharmacokinetics, independence from CYP450 bioactivation and lower impact on the inhibition of hippocampal neurogenesis, we have demonstrated that it also causes some degree of neurotoxicity, underlining the need to carefully deliberate its use, even in a context of neuropathic pain treatment.

The assays herewith presented could be complemented by further studies. Behavioral studies would clarify if the present experimental conditions imply some degree of dependence and abuse potential, which remain particularly elusive for tapentadol. Moreover, immunohistochemistry would shed light on the expression of specific tissue and cell toxicity and damage biomarkers, thereby broadening the information obtained through histological analysis. Regarding brain toxicity, molecular, metabolic, and histological analyses could be performed with samples from brain regions besides the cortex, such as the hippocampus, to ascertain whether the effects are region-specific. The dose range and the exposure period assayed could also be expanded in order to unveil eventual dose- and time-dependent outcomes, as well as to mimic overdose and chronic use situations. To clarify the putative toxicological role of active metabolites such as M1, these could be directly administered instead of the parental compounds. Likewise, the use of opioid antagonists could also elucidate the contribution of opioid receptor agonism to toxicity. Since opioids are frequently used together with other medications, combined drug exposure assays could be performed, for instance, with selective 5-HT reuptake inhibitors, tricyclic antidepressants, and monoamine oxidase inhibitors. Such an approach would clarify the possibility of toxicity exacerbation due to drug–drug interactions.

## Figures and Tables

**Table 1:** Primer nucleotide sequences and specifications of the amplification programs used for quantitative Real-Time PCR (qRT-PCR) gene expression analysis of lung, cardiac, and neurotoxicity biomarker genes.

Gene	Forward Primer (5′→3′)	Reverse Primer (5′→3′)	Annealing Temperature (°C)	No. of Amplification Cycles	Reference
CC16 (Clara cell protein-16)	CATCAGCCCACATCTACAGAC	GGGCTTTAGCGTAGAATATCT	55	35	[173]
MCP-1 (Monocyte chemoattractant protein-1)	CCCACTCACCTGCTGCTACTC	AGAAGTGCTTGAGGTGGTTGTG	55	40	[174]
MMP-7 (Matrix metalloproteinase-7)	TCGGCGGAGATGCTCACT	TGGCAACAAACAGGAAGTTCAC	50	40	[175]
SP-A (Pulmonary surfactant protein A)	TACCAGAGCAGGAGGCAACA	CAATACTTGCAATGGCCTCGTT	55	35	[176]
SP-D (Pulmonary surfactant protein D)	AAATCTTCAGGGCGGCAAA	GGCCTGCCTGCACATCTC	55	40	[176]
IL-6 (Interleukin-6)	TCCTACCCCAACTTCCAATGCTC	TTGGATGGTCTTGGTCCTTAGCC	55	40	[177]
Plau/UPA (Plasminogen activator, urokinase)	TCACTGGCTTCGGACAAGAGA	CCAATGTGGGACTGAATCCAG	55	40	[178]
TGF-β2 (Transforming growth factor-β2)	TTCAGAATCGTCCGCTTCGAT	TTGTTCAGCCACTCTGGCCTT	50	41	[179]
TIMP-1 (Tissue inhibitor of metalloproteinase-1)	TCTGGCATCCTCTTGTTGCTAT	CCACAGCGTCGAATCCTT	50	41	[180]
SNCA (α-Synuclein)	TGCTGTGGATATTGTTGTGG	AGGTGCGTAGTCTCATGCTC	55	35	[181]
BDNF (Brain-derived neurotrophic factor)	AAACGTCCACGGACAAGGCA	TTCTGGTCCTCATCCAGCAGC	55	37	[182]
GS (Glutamine synthetase)	CCACTGTCCCTGGGCTTAGTTTA	AGTGACATGCTAGTCCCACCAA	55	37	[183]
S100β (S100 calcium binding protein B)	GGGTGACAAGCACAAGCTGAA	AGCGTCTCCATCACTTTGTCCA	55	35	[184]
18S rRNA (18S ribosomal RNA)	TTCGGAACTGAGGCCATGATT	TTTCGCTCTGGTCCGTCTTG	In line with that of the target gene		[185]

## Data Availability

The data presented in this study are available in the main text.
